# The Association of Metformin, Other Antidiabetic Medications and Statins on the Prognosis of Rectal Cancer in Patients with Type 2 Diabetes: A Retrospective Cohort Study

**DOI:** 10.3390/biom12091301

**Published:** 2022-09-15

**Authors:** Sami Erkinantti, Ari Hautakoski, Reijo Sund, Martti Arffman, Elina Urpilainen, Ulla Puistola, Esa Läärä, Arja Jukkola, Peeter Karihtala

**Affiliations:** 1 Medical Research Center Oulu, Oulu University Hospital and University of Oulu, P.O. Box 8000, 90014 Oulu, Finland; 2 Department of Obstetrics and Gynecology, PEDEGO Research Unit, Medical Research Center Oulu, University of Oulu and University Hospital of Oulu, P.O. Box 23, 90029 Oulu, Finland; 3 Institute of Clinical Medicine, University of Eastern Finland, P.O. Box 1627, 70211 Kuopio, Finland; 4 Department of Public Health and Welfare Finnish Institute for Health and Welfare, P.O. Box 30, 00271 Helsinki, Finland; 5 Research Unit of Mathematical Sciences, University of Oulu, P.O. Box 3000, 90014 Oulu, Finland; 6 Department of Oncology and Radiotherapy, Tampere University Hospital, Cancer Center Tampere, Faculty of Medicine and Health Technology, Tampere University, P.O. Box 2000, 33521 Tampere, Finland; 7 Department of Oncology, Helsinki University Hospital Comprehensive Cancer Center and University of Helsinki, P.O. Box 180, 00029 Helsinki, Finland

**Keywords:** rectal, cancer, metformin, statins, diabetes, prognosis, cohort, insulin

## Abstract

Metformin and statin use have been associated with an improved prognosis for colorectal cancer in persons with type 2 diabetes (T2D). Data regarding rectal cancer (RC) have been inconclusive; therefore, we investigated the issue with high-quality data and a robust study design. We identified 1271 eligible patients with T2D and incident RC between 1998 and 2011 from the Diabetes in Finland (FinDM) database. Cox models were fitted for cause-specific mortality rates to obtain adjusted estimates of the hazard ratios (HR) with 95% confidence intervals (CI) in relation to use of antidiabetic medication (ADM) and statins before the RC diagnosis and for post-diagnostic use in a time-dependent exposure manner. No sufficient evidence was found for either pre- or post-diagnostic metformin use and RC mortality (HR 0.96, 95% CI 0.67–1.38, and 0.70, 95% CI 0.45–1.10, respectively) when compared to other oral ADMs. Both pre- and post-diagnostic statin use appeared to be inversely associated with mortality from RC (HR 0.77 95% CI 0.63–0.94, and 0.57, 95% CI 0.42–0.78, respectively). Our study was inconclusive as to the association of metformin use with the prognosis of RC, but statin use was found to predict reduced mortality, both from RC and from other causes of death in persons with T2D.

## 1. Introduction

Rectal cancers (RC) are the eighth most common cancers diagnosed globally, with the tenth highest mortality [1]. Type 2 diabetes (T2D) is also a growing global concern, with over 450 million cases occurring in 2017 [2]. T2D has been associated with RC incidence [3] and mortality [4]. Metformin is a first-line T2D medication with observed preclinical anticancer effects [5]. The use of metformin in patients with T2D has been associated with improved colorectal cancer (CRC) survival [6,7,8] in some meta-analyses, although the findings have been variable [9].

Statins are a lipid-lowering medication commonly used by persons with T2D to decrease the risk of cardiovascular disease [10]. Statin use has been associated with a small survival benefit in patients with T2D and CRC [11], increasing with cumulative exposure. There is also preclinical evidence of the antitumor effects of statins in vitro [12,13].

There are fundamental differences in the pathogenesis and biology of RCs and cancers of the colon (CC) [14,15,16]. Therefore, we decided to focus solely on RCs, and to our knowledge, this is one of the first studies to do so.

This study was a retrospective national cohort study that included Finnish persons with T2D. We examined the association of RC prognosis with both pre- and post-diagnostic use of metformin, other antidiabetic medications, and statins in RC patients with T2D by using a robust study design and data from multiple high-quality Finnish registers. The aim was to obtain further evidence concerning the hypothesized association between metformin and statins with the prognosis of RC.

## 2. Materials and Methods

We followed the Strengthening the Reporting of Observational Studies in Epidemiology (STROBE) guidelines for observational studies in writing this article [17]. Patient data were obtained from the Diabetes in Finland (FinDM) database [18], which has been set up for epidemiological monitoring of diabetes in Finland. The database combines data from multiple national registers, including the Care Register for Health and the Hospital Discharge Register from the Finnish Institute for Health and Welfare, the Special Reimbursement Register and the Prescription Database from the Social Insurance Institution of Finland, and the Cause of Death Register from Statistics Finland. These registers have enabled an accurate assessment of the purchased drugs since 1994. FinDM classifies the diagnosis of diabetes using either hospital records (since 1969 for inpatients, and since 1998 for outpatients) or on entitlement for elevated reimbursement for antidiabetic medication (ADM) since 1964, or on the purchase of reimbursed ADM. Categorization between type 1 diabetes and T2D is based on the classification of the FinDM database. FinDM defines diabetes type by combining multiple data sources, including medication reimbursement and diagnosis data from comprehensive hospital and primary care visit registers [18]. Persons with gestational or unspecifiable diabetes were excluded. There was a significant group of persons classified as type 2 diabetics in the age group 1–40 years, and we decided to exclude them to eliminate the risk of bias from misclassification. FinDM has been confirmed to have good coverage in a study that compared it to a local register in Southern Finland [19]. The linkage of data is based on a unique personal identification code given at birth or when included in the Finnish social security system, rendering individuals easily trackable.

The data regarding cancer cases were obtained from the Finnish Cancer Registry (FCR) and linked with data from the FinDM. The FCR contains data from almost all cancer cases diagnosed in Finland since 1953, including the date of diagnosis, histology, and morphology, with an estimated 96% [20] completeness of the records for solid tumors. The FCR receives follow-up data from registers maintained by Statistics Finland, including dates and causes of deaths. Experts at the FCR compare these data to all available clinical data concerning the patient’s cancer case and judge whether the deaths are cancer related. Our analysis of cause specificity is based on this judgment.

**Figure 1:**
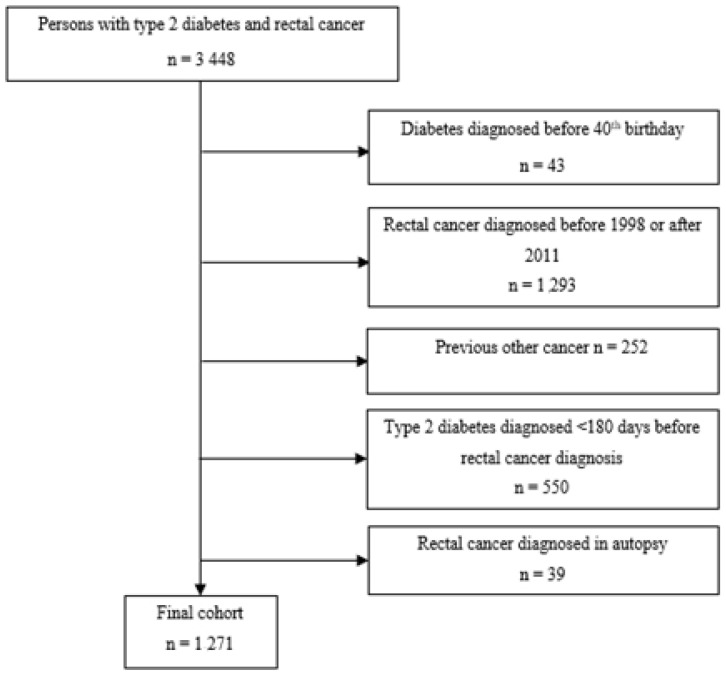
Flowchart of the cohort selection process.

The cohort selection is presented in the flowchart in Figure 1. There were 3448 persons diagnosed with T2D and RC. Persons with T2D diagnosed prior to their 40th birthday were excluded to minimize the inclusion of T1D cases mislabeled as T2D due to T1D being likelier for persons under 40 years, and due to a high proportion of hereditary cancer syndromes in this age group. RC cases diagnosed before 1998 or after 2011 were excluded, as were persons with another previous cancer diagnosis except non-melanoma skin cancers based on the International Classification of Diseases for Oncology (ICD-O-3) codes C44 plus M-8090-8095/3, M-8097-8098/3, M-8102/3 and M-8110/3, persons with T2D diagnosed <180 days before the RC diagnosis, and RC cases diagnosed at autopsy. The cases were followed until the end of 2013. The final cohort contained 1271 individuals. RC was defined as a diagnosis by the International Classification of Diseases 10th Revision (ICD-10) codes C19 (malignant neoplasm of rectosigmoid junction) and C20 (malignant neoplasm of rectum), ICD-O-3 codes C19.9 and C20.9, and morphology code M-8140/3. The decision to include both C19 and C20 under the definition of RC was based on the classification of RC by the GLOBOCAN Database [21] and the Finnish Cancer Registry.

Persons in the cohort were divided into five mutually exclusive groups according to their ADM usage during the three years preceding the diagnosis of RC: (1) metformin only, (2) other oral ADM only, (3) metformin and other oral ADM, (4) insulin, and (5) no history of regular ADM use. Statin use was classified independently of the use of ADM into users and non-users. Cumulative medication use was assessed as defined daily doses (DDD) for three years prior to the cancer diagnosis. The criterion for oral ADM and statin usage was the purchase of the medication for at least 180 days or longer during the three years prior to the cancer diagnosis. Purchase periods of ADM or statin less than 180 days prior to the diagnosis placed the person in the group of “no history of regular ADM use” or “statin non-user”, respectively. At least one purchase of insulin was sufficient to categorize a patient as belonging to the insulin user group.

The FCR records the stage of cancer into the following classes: Localized, regionally spread, distantly spread, and unknown. In our study, we have relabeled “localized” and “regionally spread” cancers as “non-metastasized” and those with distant spread as “metastasized” [20]. Non-metastasized cases include tumors that have grown only locally or to adjacent tissues but have not metastasized to regional or distant lymph nodes (Stages 0, IA, IIA, IIB, IIIB, and TNM Tis-4N0M0) [22]. Advanced cases include tumors that have metastasized to regional or distant lymph nodes, with, or without local advancement to nearby tissues, corresponding to stage and TNM T1-4N0-3M0-1 with N and/or M ≥ 1. Unknown cases have no reliable staging information available.

## 3. Statistical Methods

We analyzed mortality from RC and from other causes of death, respectively, in relation both to the medications used before cancer diagnosis and post-diagnostic medications. In the former analyses, the follow-up started on the date of RC diagnosis and ended on the date of death, emigration, or closing of the follow-up on 31 December 2013. The Aalen–Johansen estimator of the cumulative incidence function for competing risks was used to describe cumulative mortality from RC and from other causes of death in the different pre-diagnostic ADM groups, as well as among users and non-users of statins. The cause-specific mortality rates were analyzed by Cox proportional hazard models to obtain estimated hazard ratios (HR) with 95% confidence intervals (CI), adjusting for the confounding effects of age, year, duration of diabetes, and RC stage. A possible nonlinear dose-dependent effect of the medications was assessed by replacing the medication group indicators in the Cox models with cubic spline terms for the total amount of DDDs per medication group.

The association between post-diagnostic medication use and mortality from RC and other causes of death was analyzed using time-dependent Cox regression models. In these analyses, we only included those patients from the original cohort who were still alive and under follow-up on the date when one year had passed since cancer diagnosis. The follow-up also started on that date. Exposure to medication was recorded monthly, starting from the date of RC diagnosis. Exposure to metformin, other oral ADMs, and statins was each represented as a time-dependent binary indicator variable (use vs. non-use) according to the following criteria for being exposed to a given medication at any month: an exposure period of at least 180 days after RC diagnosis was required, and exposure was defined to end 270 days after the final purchase of medication. Regarding insulin, two purchases were enough for the person to be categorized as an “insulin user” until the end of the follow-up. These time intervals were partly based on the Finnish medical reimbursement system, which encourages three-month medication purchases. The following variables were included in the model: statin, metformin, other oral ADM, insulin use, sex, stage, diabetes duration, current age, and year of diagnosis. As we were specifically interested in the contrast between metformin use and the use of other oral ADMs, we also derived from the pertinent models the point estimates of the HR (with 95% CIs) associated with this contrast.

All statistical analyses were performed with the R environment (version 4.1.2, R Core Team. R: A Language and Environment for Statistical Computing. R Foundation for Statistical Computing: Vienna, Austria, 2020. Available online: https://www.R-project.org/, accessed on 20 September 2020) [23]. The functions in the “survival” package of R functions were used to compute the Aalen–Johansen estimators of cumulative mortality by cause to fit the Cox models and to diagnose possible deviations from the underlying model’s assumptions [23,24]. Missing data were encountered only concerning the spread of the cancer, and we labeled these cases “unknown” spread.

## 4. Results

Information regarding the study cohort is presented in Table 1.

The total number of RC cases was 1271, and the median follow-up time was 2.8 years. Most RCs were in the age groups of 70–74 years and 75–79 years, with a median age at RC diagnosis of 74, reflecting the disease burden on the elderly population. There were more male patients in the cohort, and there were more metformin and statin users in the male group than in the female group.

Metformin users were slightly younger, had shorter median diabetes duration, and had metastasized disease more often when compared to the reference group of other oral ADM users. Insulin users had the longest median diabetes duration and the highest proportion of metastasized RC of all five study groups.

Statin users and non-users had a similar median age at RC diagnosis and comparable median diabetes duration. There was a slightly higher proportion of metastasized RCs in the statin users than in the non-users.

The most used pre-diagnostic oral ADMs, other than metformin, were sulphonylureas, comprising 93% of the other oral ADM group (Supplementary Table S1). The most frequently used statins in the cohort were simvastatin (81% of users) and atorvastatin (38% of statin users) (Supplementary Table S2).

**Figure 2:**
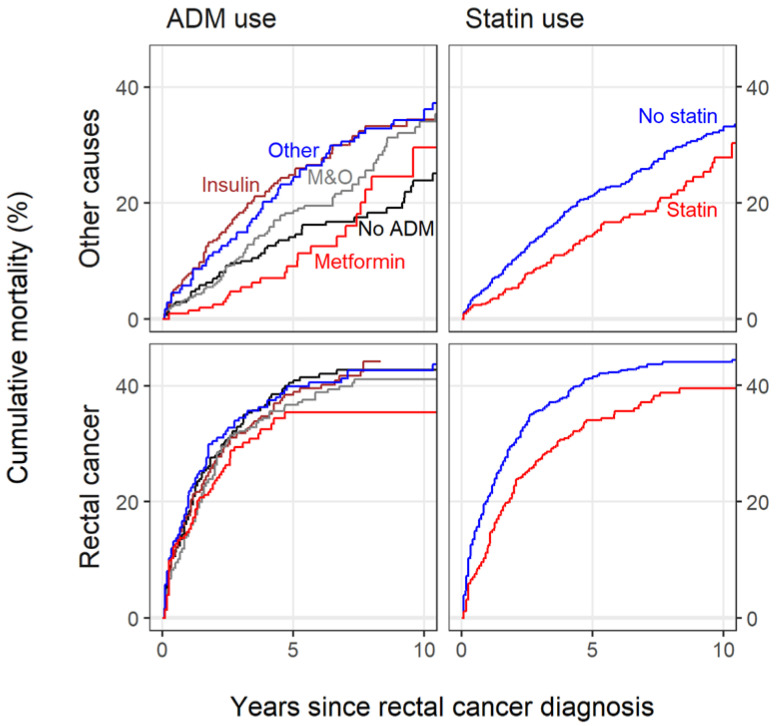
Cumulative mortality from rectal cancer and from other causes in the different pre-diagnostic medication groups. Antidiabetic medication (ADM): Red = metformin, blue = other oral ADM, brown = insulin, gray = metformin and other oral ADM, and black = no history of regular ADM use. Statin: Blue = non-users; red = users.

Cumulative mortality from RC and other causes of pre-diagnostic use are presented in Figure 2. Metformin users had a less steep mortality curve when compared to the other ADM groups. A clear difference between statin users and non-users can be observed for both causes.

The total number of deaths from different causes and HR estimates with 95% CIs are presented in Table 2.

**Figure 3:**
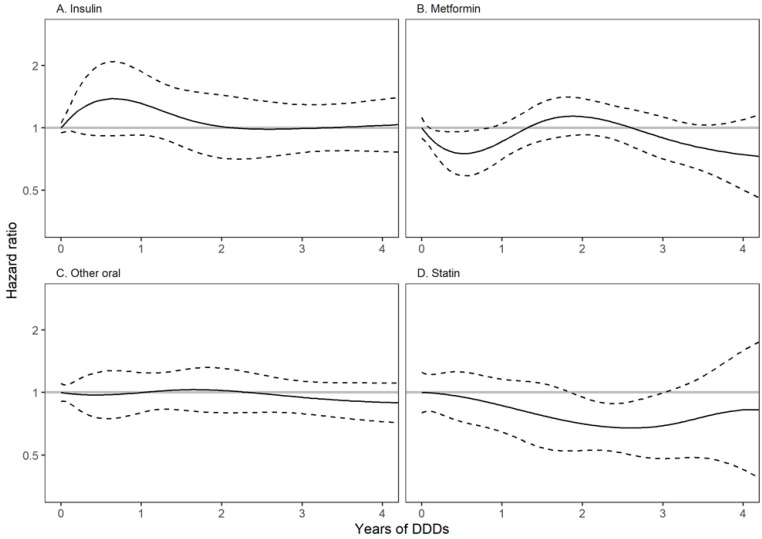
Estimated hazard ratios (with pointwise 95% confidence limits) of rectal cancer (RC) mortality by cumulative defined daily dose amount of antidiabetic medications and statins during the three years preceding RC diagnosis in selected medication groups. (**A**) = insulin, (**B**) = metformin, (**C**) = other oral ADM and (**D**) = statin. Solid line represents hazard ratio, with dashed lines indicating 95% confidence intervals.

There was no evidence for an association between pre-diagnostic metformin use and RC mortality (HR 0.96, 95% CI 0.67–1.38) when compared to use of other oral ADM. For other causes of death, the mortality in metformin users was found to be lower than in users of other oral ADM, but not conclusively so (HR 0.66, 95% CI 0.40–1.09). Statin use was found to be associated with reduced mortality from RC and from other causes (HR 0.77, 95% CI 0.64–0.94, and 0.68, 95% CI 0.53–0.88). No consistent trend was observed in the DDD analysis for an association between the cumulative use of any medication and RC mortality (Figure 3).

Of the original cohort, 867 patients survived the first year after the diagnosis of RC. The analyses of mortality in relation to use of post-diagnostic medications comprised these patients only, their follow-up starting one year after cancer diagnosis. The results concerning post-diagnostic use vs. non-use of the medications of interest with mortality from RC and from other causes of death are reported in Table 3. The contrast between the use of metformin and that of other oral ADMs, although pointing in the same direction as with pre-diagnostic use, had wider error margins and were inconclusive for mortality from both RC (HR 0.70; 95% CI 0.45–1.10) and other causes of death (HR 0.88; 0.55–1.41). For post-diagnostic statin use, evidence was found for an association with reduced RC mortality (HR 0.57; 95% CI 0.42–0.78) and mortality from other causes (HR 0.52, 95% CI 0.38–0.71), which is concordant with that for pre-diagnostic use. When analyzing the mortality from the two causes in relation to cumulative use of any of the medications, no evidence for any monotonic trend could be found (data not shown).

## 5. Discussion

In this study, which focused on survival from RC among patients with preexisting T2D, statin use was observed to have an inverse association with mortality from RC and other causes. There was no sufficient evidence of an association between the studied ADMs and RC mortality.

Several epidemiological meta-analyses [7,8,25,26] and one additional study not included in the previously mentioned meta-analyses [27] have associated metformin use in patients with T2D and CRC with improved overall and disease-specific mortality. There has been an association in T2D patients who use metformin and who have undergone radical RC resection and subsequent chemoradiotherapy with either improved pathologic complete response and cancer-specific mortality [28,29] or no difference in the outcomes [30,31], when compared to metformin non-user patients with T2D. Our epidemiological study was one of the first to focus solely on RC in the general diabetic population, and we did not find convincing evidence about the association between metformin use, either pre-diagnostic or post-diagnostic, and reduced mortality from RC. A similar result was observed in a previous study analyzing post-diagnostic use and RC mortality [32]. Additionally, a trend toward lower association, especially in women, has been reported [33], and higher metformin adherence has been previously associated with lower RC mortality when compared to low adherence [34]. A single study using a similar methodology to ours found no association between post-diagnostic metformin use and RC mortality [35].

The mechanism of action for metformin is not completely understood, but it involves AMP-activated protein kinase (AMPK) activation and the inhibition of mitochondrial respiration [36]. Inhibition of mitochondrial respiration might occur only with suprapharmacological doses used in vitro [37,38]. Metformin has both direct and indirect anticancer effects in preclinical studies [39]. Direct effects include AMPK-dependent cell-cycle arrest, stabilization of p53, and inhibition of the mammalian target of the rapamycin (mTOR) pathway. Indirect effects include reduced blood glucose levels, improved insulin status, and a decrease in proinflammatory cytokines.

Hyperinsulinemia is present in T2D due to insulin resistance, and supraphysiological levels are hypothesized to amplify insulin’s mitogenic effect, both through direct receptor activation and an increase in IGF-1 signaling [40], promoting the proliferation of malignant cells. There are two forms of the insulin receptor (INSR), A and B, of which INSR-A handles the mitogenic effects of insulin, while INSR-B handles glucose uptake [41]. INSR-A also binds IGFs and proinsulin, and it is overexpressed in various malignant cells. Its activation enhances malignant transformation by promoting mitogenic signaling, a possible mechanism for the link between various cancers and hyperinsulinemia. Elevated blood glucose levels have been associated with an increased risk for both incident and fatal cancer dose-dependently, although the ultimate etiological factor (dyslipidemia, insulin resistance and/or increased proinflammatory cytokines) is still unclear [41]. Exogenous insulin therapy with synthetic insulin analogs may lead to circulating insulin levels of up to 10–50 times higher [40] than physiologic levels. Previous studies have found mixed results between the association of disease-specific mortality and insulin use in patients with T2D and CRC [42,43]. Our results are in line with the former, even though persons in the insulin users group had the longest median duration of diabetes and the highest proportion of metastasized RCs.

The other most used oral ADM group in our study was sulphonylureas, which induces glucose-independent insulin release from pancreatic B-cells [44], thus possibly leading to hyperinsulinemia. There has been no association between either ever-use [44] or post-diagnostic use [35] of sulphonylureas and disease-specific mortality from RC in patients with T2D. We used other oral ADM users as a reference group to eliminate bias that would be introduced by comparing medication users to people who do not use any medications, when calculating pre-diagnostic medication use. There were a large number of other oral ADM medication groups, each with a relatively small number of patients. Therefore, we considered it not useful to employ such a detailed categorization in our statistical analysis.

Statin use was observed to be associated with an improved prognosis of RC and reduced mortality from other causes, both for pre- and post-diagnostic use. These findings are similar to those of three previous epidemiological studies conducted in patients with CRC and T2D [11,26,45]. Studies concerning RC and statin use in nondiabetics have linked statin use with reduced postoperative mortality [46], improved response to neoadjuvant therapy [47,48] and improved overall and disease-specific survival [49]. A recent meta-analysis associated pre-diagnostic statin use with reduced CRC mortality and reduced overall recurrence, but not with overall progression-free survival, regardless of subsite [50]. Multiple ongoing clinical trials have investigated statin use as an adjunct to neoadjuvant therapy [51,52]. We compared statin users to non-users because no comparable treatment group of persons taking other lipid-lowering medications was available. This is a possible source of healthy user bias.

In vitro studies of statins have found that they inhibit the cell cycle, induce cancer cell apoptosis through intrinsic and extrinsic pathways, and inhibit cholesterol synthesis [12]. Cancer cells have a high requirement for cholesterol and isoprenoids; therefore, this could be the mechanism behind the beneficial effect of statins, as seen in clinical and epidemiological studies. Hydrophilic statins accumulate mainly in the liver, whereas hydrophobic statins also penetrate extrahepatic tissues and may therefore have a greater anticancer effect. Most persons in our cohort used hydrophobic statins.

Limitations of our study include a lack of information regarding body mass index and glycated hemoglobin, which indicate the severity of T2D and can influence prognosis. We based our study on register data that had limited information about socioeconomic status, lifestyle factors, and other simultaneous diseases, leaving the possibility of confounding due to unmeasured factors. The register data had no information about the medication use of patients in long-term institutionalized or hospital care, although this forms only a small portion of persons with T2D. Because the classification of cancer stage based on data from the Finnish Cancer Registry was relatively crude, residual confounding by stage might be present if persons using only metformin on average had an earlier stage disease compared to those using other oral ADM and/or insulin. The database also contains no reliable data about the treatment modalities for the cancer cases and no data on clinical complications. All these shortcomings may contribute to confounding by indication of an unknown amount to the results concerning the effects of post-diagnostic medications, especially since it is possible that the use of ADMs and/or statins is associated with those variables that are prognostically important but for which data are missing. Our median follow-up time was relatively short due to the high proportion of metastasized RCs in our cohort. However, the proportion of metastasized cases and the follow-up time were similar in another Finnish study concerning diabetes and CRC [27].

The strengths of this study are its large cohort from high-quality registers and a robust study design, including post-diagnostic medication use. The data from Finnish registers are comprehensive, high quality, and reliable [20,53,54], thus providing accurate information regarding medication use, cause of death, and individual identification. We had a relevant number of metformin and statin users for the statistical analysis, and we had DDD data to examine the association between the amount of medication used and HR.

## 6. Conclusions

We found evidence for the beneficial prognostic association of pre- and post-diagnostic statin use in RC patients with T2D, but no evidence for an association between any of our studied ADM and RC survival was found.

## Figures and Tables

**Table 1:** Characteristics of the study cohort in different pre-diagnostic antidiabetic medication (ADM) groups and by statin use.

	Antidiabetic Medication (ADM)					Statins		
	Metformin (%)	Other Oral ADM (%)	Metformin and Other oral ADM (%)	Insulin (%)	No History of Regular ADM Use (%)	Yes (%)	No (%)	Total (%)
Number of patients	203 (100)	174 (100)	326 (100)	296 (100)	272 (100)	541 (100)	730 (100)	1271 (100)
Sex								
Male (%)	127 (63)	96 (55)	186 (57)	187 (63)	166 (61)	342 (63)	420 (58)	762 (60)
Female (%)	76 (37)	78 (45)	140 (43)	109 (37)	106 (39)	199 (37)	310 (42)	509 (40)
Age groups (years)								
41–59	18 (9)	8 (5)	24 (7)	29 (10)	22 (8)	41 (8)	60 (8)	101 (8)
60–64	23 (11)	8 (5)	50 (15)	30 (10)	28 (10)	68 (13)	71 (10)	139 (11)
65–69	36 (18)	23 (13)	44 (13)	53 (18)	63 (23)	106 (20)	113 (15)	219 (17)
70–74	47 (23)	34 (20)	65 (20)	60 (20)	60 (22)	130 (24)	136 (19)	266 (21)
75–79	35 (17)	35 (20)	81 (25)	66 (22)	39 (14)	108 (20)	148 (20)	256 (20)
80–84	28 (14)	30 (17)	39 (12)	39 (13)	32 (12)	56 (10)	112 (15)	168 (13)
85–96	16 (8)	36 (21)	23 (7)	19 (6)	28 (10)	32 (6)	90 (12)	122 (10)
Median age at RC diagnosis	73	77	74	73	72	72	74	74
Interquartile range	66–79	71–83	66–79	67–79	67–79	66–78	68–81	67–79
Duration of diabetes (years)								
0.50–<3	86 (42)	46 (26)	22 (7)	8 (3)	99 (36)	99 (18)	162 (22)	261 (21)
3–<6	66 (33)	64 (37)	74 (23)	21 (7)	45 (17)	110 (20)	160 (22)	270 (21)
6–<12	39 (19)	50 (29)	157 (48)	113 (38)	69 (25)	183 (34)	245 (34)	428 (34)
≥12	12 (6)	14 (8)	73 (22)	154 (52)	59 (22)	149 (28)	163 (22)	312 (25)
Median	3.4	4.7	8.0	12.2	5.4	7.7	7.0	7.3
Interquartile range	2.1–6.0	2.8–7.5	5.5–11.3	8.8–16.1	1.4–11.1	3.9–12.4	3.3–11.2	3.6–11.9
Cancer stage								
Non-metastasized	68 (33)	64 (37)	114 (35)	99 (33)	96 (35)	187 (35)	254 (35)	441 (35)
Metastasized	100 (49)	69 (40)	158 (48)	152 (51)	124 (46)	281 (52)	322 (44)	603 (47)
Unknown	35 (17)	41 (24)	54 (17)	45 (15)	52 (19)	73 (13)	154 (21)	227 (18)
Outcome at the end of the follow-up								
RC death	65 (32)	75 (43)	125 (38)	117 (40)	110 (40)	181 (33)	311 (43)	492 (39)
Death from other causes	25 (12)	62 (36)	86 (26)	84 (28)	58 (21)	94 (17)	221 (30)	315 (25)
Alive	113 (56)	37 (21)	115 (35)	95 (32)	104 (38)	266 (49)	198 (27)	464 (37)

**Table 2:** Numbers of deaths (n) by cause and estimated hazard ratios (HR) with 95% confidence intervals (CI) related to mortality from rectal cancer (RC) and from other causes by sex, year of diagnosis, age, stage, antidiabetic medication (ADM), and the use of statins during the three years before the diagnosis of RC. The estimation results are based on Cox regression models, including all variables.

	Death from Rectal Cancer			Death from Other Causes		
	n	Hazard Ratio ^c^	95% Confidence Interval	n	Hazard Ratio ^c^	95% Confidence Interval
Sex						
Male	290	1.07	(0.88–1.29)	186	1.76	(1.38–2.25)
Female	202	1.00	Reference	129	1.00	Reference
Year of diagnosis						
1998–2002	165	1.00	Reference	135	1.00	Reference
2003–2007	180	0.86	(0.69–1.07)	128	1.25	(0.96–1.63)
2008–2011	147	0.68	(0.52–0.87)	52	0.85	(0.58–1.25)
Age group (years)						
41–59	33	0.73	(0.49–1.09)	8	0.21	(0.10–0.45)
60–64	47	0.67	(0.48–0.95)	15	0.33	(0.19–0.58)
65–69	74	0.89	(0.66–1.21)	51	0.89	(0.61–1.30)
70–74	104	1.00	Reference	60	1.00	Reference
75–79	102	1.19	(0.90–1.56)	73	1.71	(1.21–2.42)
80–84	71	1.51	(1.11–2.05)	63	2.86	(1.98–4.12)
85–97	61	2.15	(1.55–2.99)	45	4.37	(2.87–6.65)
Duration of diabetes (years)						
0.5–<3	103	1.00	Reference	55	1.00	Reference
3–<6	102	1.00	(0.75–1.33)	58	0.97	(0.66–1.43)
6–<12	161	0.99	(0.75–1.29)	119	1.40	(0.98–1.98)
≥12	126	0.94	(0.70–1.26)	83	1.16	(0.78–1.73)
Cancer stage						
Non-metastasized	70	1.00	Reference	153	1.00	Reference
Metastasized	330	5.57	(4.28–7.25)	87	0.86	(0.66–1.14)
Unknown	92	3.01	(2.20–4.12)	75	1.22	(0.92–1.61)
ADM ^a^						
Other oral ADM	75	1.00	Reference	62	1.00	Reference
Metformin	65	0.96	(0.67–1.38)	25	0.66	(0.40–1.09)
Metformin and other oral ADM	125	1.03	(0.76–1.40)	86	1.01	(0.71–1.44)
Insulin	117	1.15	(0.83–1.58)	84	1.53	(1.04–2.25)
No regular history of ADMuse ^b^	110	1.05	(0.77–1.43)	58	0.75	(0.52–1.09)
Statin use ^a^						
No	311	1.00	Reference	221	1.00	Reference
Yes	181	0.77	(0.63–0.94)	94	0.68	(0.53–0.88)

^a^ Medication duration >180 days except for insulin, which is classified as user or non-user. ^b^ No history of regular ADM use. ^c^ Adjusted for the effects of age, year, duration of diabetes, and stage.

**Table 3:** Hazard ratios and 95% confidence intervals (in parentheses) for post-diagnostic cancer-specific mortality and mortality from other causes in relation to medication. ADM = antidiabetic medication, RC = rectal cancer, HR = hazard ratio, 95% CI = 95% confidence interval.

	Death from RC		Death from Other Cause	
	n * HR	95% CI	n * HR	95% CI
Metformin	67 0.56	(0.42–0.76)	66 0.67	(0.49–0.92)
Other oral ADM	75 0.80	(0.60–1.07)	71 0.76	(0.56–1.03)
Insulin	64 0.82	(0.60–1.13)	79 1.59	(1.17–2.16)
Statin	65 0.57	(0.42–0.78)	67 0.52	(0.38–0.71)

* Number of deaths when being exposed to the pertinent medication.

## Data Availability

The data presented in this study are available on request from the corresponding author. The data are not publicly available for ethical reasons.

## Supplementary Materials

The following supporting information can be downloaded at: https://www.mdpi.com/article/10.3390/biom12091301/s1, Table S1: Other oral ADM subgroups; Table S2: Statin subgroups.
